# Physicochemical Properties and Oxidative Stability of an Emulsion Prepared from (-)-Epigallocatechin-3-Gallate Modified Chicken Wooden Breast Myofibrillar Protein

**DOI:** 10.3390/antiox12010064

**Published:** 2022-12-28

**Authors:** Ke Wang, Yan Li, Yimin Zhang, Jingxin Sun

**Affiliations:** 1 College of Food Science & Engineering, Qingdao Agricultural University, Qingdao 266109, China; 2 College of Food Science & Engineering, Shandong Agricultural University, Tai’an 271018, China; 3 Shandong Research Center for Meat Food Quality Control, Qingdao Agricultural University, Qingdao 266109, China

**Keywords:** wooden breast, myofibrillar protein, emulsion, (-)-epigallocatechin-3-gallate, physicochemical properties, oxidative stability

## Abstract

The deterioration of wooden breast myofibrillar protein (WBMP) causes a decline in its processing performance, and the protein becomes easier to oxidize. Previous studies have revealed that the use of (-)-epigallocatechin-3-gallate (EGCG) may improve the physicochemical properties and oxidative stability of proteins in aqueous solutions. The effects of varying concentrations (0.01%, 0.02%, 0.03%, and 0.04% *w*/*v*) of EGCG on the physicochemical properties of a WBMP emulsion (1.2% WBMP/10% oil) and the inhibition of lipid and protein oxidation were studied. The results revealed that a moderate dose of EGCG (0.03%) could significantly (*p* < 0.05) improve the emulsion activity index (4.66 ± 0.41 m^2^/g) and emulsion stability index (91.95 ± 4.23%), as well as reduce the particle size of the WBMP emulsion. According to the micrographs and cream index, 0.03% EGCG retarded the phase separation by stopping the aggregation of droplets and proteins, thus significantly improving the stability of WBMP emulsions. During storage at 50 °C for 96 h, 0.03% EGCG inhibited lipid oxidation (lipid hydroperoxide and 2-thiobarbituric acid-reactive substance formation) and protein oxidation (carbonyl formation and sulfhydryl loss). In contrast, lower and higher EGCG concentrations (0.01%, 0.02%, and 0.04%) demonstrated shortcomings (such as weak antioxidant capacity or protein over-aggregation) in improving the quality and oxidation stability of the emulsion. In conclusion, a moderate dose of EGCG (0.03%) can be used to improve the quality and shelf life of WBMP emulsions.

## 1. Introduction

Wooden breast (WB) is a kind of abnormal meat that has negatively impacted the poultry industry [1]. WB meat shows obvious hardening on the upper part of the pectoralis major muscles and is often accompanied by stripes and bleeding spots [2]. According to an existing survey, the incidence of WB is more than 60% worldwide, and serious cases account for a large proportion [3]. Myofibrillar protein (MP) as the main protein component [4], is also severely affected by WB and influenced its functional properties (such as its emulsifying property) [5]. MP stabilizes the fat and water in meat products and also acts as an emulsifier, which is very beneficial to the manufacture of emulsions [6]. Emulsions are widely used in the food industry and often have specific functions such as wind flavor carriers or functional substances [7]. Oil-in-water (O/W) emulsions are most commonly used to package chemically unstable hydrophobic active substances in the food industry [8]. These emulsions are composed of emulsified oil droplets dispersed in a continuous phase. Therefore, it is necessary to add stabilizers, including amphiphilic polymers and solid particles, to prevent phase separation between the oil and water. Emulsifiers can be adsorbed at the oil–water interface, and MP can prevent lipid droplet oxidation due to its ability to scavenge free radicals, chelate metal ions, and/or form steric hindrances [9]. In addition, MP can be used as a carrier in emulsion systems for nutritional and bioactive components.

An emulsion is a thermodynamically unstable system that is easily destroyed over time. The destruction steps include sedimentation, flocculation, aggregation, and Ostwald maturation, which result in phase separation [10]. The amphiphilic biopolymers (protein) gather at the oil–water interface to form an interface coating during the homogenization process, which protects the droplets from flocculation and coalescence through a combination of spatial and electrostatic repulsion interactions [11]. However, the decline in MP quality caused by WB has led to the deterioration of the emulsifying properties and stability of the emulsion. Unsaturated fatty acids in emulsions are easily oxidized by light, oxygen, and heat. It is difficult to control the quality of emulsions during transportation, storage, and retail [7]. In our previous research, we found that the emulsifying performance of moderate and severe WBMP was worse than that of normal MP, and it was more vulnerable to the adverse effects of oxidation [5]. Improving the physicochemical properties and oxidative stability of WBMP emulsions is a key approach for the utilization of WB meat.

(-)-Epigallocatechin-3-gallate (EGCG) is the main component of tea catechins. EGCG is able to scavenge free radicals and chelate iron ions [12]. EGCG has antioxidant, anti-inflammatory, and anti-cancer effects. Therefore, it is widely used in beverages and other food industries [13]. Many studies have applied EGCG to emulsion products to ameliorate its emulsification and oxidation resistance [13,14,15]. More importantly, the interaction between proteins and polyphenols affects the structural and functional properties of proteins. The addition of polyphenols can reduce the number of protein α-helices and change the interface properties of proteins [7]. Functional group hiding and MP aggregation caused by the combination of EGCG and MP affected the emulsifying performance of meat proteins. EGCG can act as a chain-breaking antioxidant by providing a hydrogen atom from its hydroxyl group to the free radicals. They also play an antioxidant role by chelating metal ions. These results confirm that EGCG has a positive effect on emulsion products. However, there is a limit to the amount of EGCG that can be added to positively affect emulsion, it has been proved by researchers that a high level (0.04%) of tea polyphenols (the main component is EGCG) has a poor effect on inhibiting lipid oxidation and actually promotes protein oxidation [16]. Furthermore, the high price of EGCG also requires producers to use it within a proper range. In brief, there are no reports of EGCG on the emulsifying properties, physical stability, and oxidative stability of WBMP, and the suitable dosage of EGCG for this process still needs to be explored. It is a core concept to improve the emulsifying performance of WBMP by combining EGCG with protein so as to form more valuable emulsion products. In this process, we want to determine the proportion relationship between EGCG and WBMP to facilitate the wide application of this strategy. Therefore, we used WBMP as a model protein emulsifier to prepare emulsions. The purpose of this study was to (1) study the effect of different doses of EGCG on the physicochemical properties of WBMP emulsions, and (2) clarify the influence of EGCG on the physicochemical and oxidative stability of WBMP emulsions during storage. Our research provides valuable insights into the improvement of the emulsion quality of inferior proteins, similar to WBMP. In addition, we hope that these works can provide some reference for elucidating the mechanism of EGCG in improving the emulsifying performance of protein-based emulsifiers.

## 2. Material and Methods 

### 2.1. Materials

WB meat was collected from Zhucheng Waimao Co. Ltd. (Zhucheng, China). WB meat was selected using visual and pressing tests based on the method described by Cai, Shao, Chen, Campbell, Nair, Suman, Beach, Guyton and Schilling [17]. Samples with medium-to-severe WB were selected for this study. Pure soybean oil was purchased from Aladdin (Shanghai, China). (-)-Epigallocatechin-3-gallate was obtained from Sigma-Aldrich Co. (St. Louis, MO, USA). All other chemicals were of analytical reagent grade.

### 2.2. Extraction of WBMP

Protein extraction was carried out following the method described by Han, Wang, Xu and Zhou [18]. The muscles were ground and homogenized at 10000 × g for 30 s in a four-buffer system (100 mmol/L KCl, 2 mmol/L MgCl_2_, 1 mmol/L EGTA, and 10 mmol/L K_2_HPO_4_; pH 7.0; chicken: buffer = 1:4 [*w*/*v*]). This process was repeated thrice. Bovine serum albumin (BSA) was used as a standard to evaluate protein concentration according to the Biuret method. Fresh proteins were stored at 4 °C for subsequent dissolution using phosphate buffer (0.6 mol/L KCl, 0.01 mol/L KH_2_PO_4_, pH 6.0) and concentration adjustment. 

### 2.3. Preparation and Storage of WBMP Emulsions

A slightly modified version of the method described by Gao, Zhao, Li, Bassey, Bai, Ye, Deng and Zhou [9] was used. WBMP was dispersed in a phosphate buffer (0.6 mol/L KCl, 0.01 mol/L KH_2_PO_4_, pH 7.0) and then hydrated by stirring at 4 °C (the solution contains 1.2% WBMP). Initially, EGCG (0.01%, 0.02%, 0.03%, or 0.04% *w*/*v* based on the final emulsion) was dissolved in 1 mL of ethanol. The WBMP solution (180 mL each) and soybean oil (20 mL) were added to a beaker containing EGCG, homogenized with an ice bath homogenizer at 15,000 rpm for 40 s, and homogenized thrice to obtain a uniform emulsion. The pH of the crude emulsion was adjusted to 7.0 with 0.1 mol/L HCl and 0.1 mol/L NaOH. The composition of the final emulsion was 1.2% *w*/*v* WBMP, 10% *w*/*v* soybean oil, and 0%, 0.01%, 0.02%, 0.03%, or 0.04% EGCG. Finally, 0.04% sodium azide was added to the emulsion to inhibit the growth of microorganisms. All the samples were incubated in the dark at 50 °C for 0, 48, and 96 h to accelerate the oxidation of the emulsion. The 0% EGCG group was used as the control.

### 2.4. Emulsion Activity Index and Emulsion Stability Index 

The emulsion activity index (EAI) and emulsion stability index (ESI) were measured using a previously reported method [19]. The emulsion (50 μL) was added to 5 mL of 1 g/L sodium dodecyl sulfate (SDS) solution. The absorbance at 500 nm was measured at 0 (A_0_) and 10 min (A_10_) after emulsion formation. The EAI and ESI values were calculated using Equations (1) and (2):

(1) $$\mathrm{EAI}\left(m^{2}/g\right)=\frac{2\times 2.303}{C\times (1-\phi )\times 10}{\times A}_{0}\times \mathrm{dilution}\mathrm{ratio}$$

(2) $$\mathrm{ESI}\left(\%\right)=\frac{A_{10}}{A_{0}}\times 100$$

where C: sample concentration (mg/mL) before emulsification, ϕ: oil volume fraction (*v*/*v*) of the emulsion (0.1), and the dilution factor is 100.

### 2.5. ζ-Potential Measurement

The emulsion sample was diluted 100 times with deionized water. The ζ-potential was measured by a Zetasizer (ZEN 3690, Malvern Instruments Inc., Malvern, UK). Collect the data before 60 s of sample balance in a DTS-7010 capillary tube at 25 °C and obtain at least 5 sequential readings [9].

### 2.6. Particle Size Distribution 

The emulsion sample was diluted 100 times with deionized water by Chen, Zou, Han, Pan, Xing, Xu and Zhou [20]. The sample was transferred to a 1 cm path length quartz cuvette subjected to dynamic light scattering (DLS) using a DLS instrument (ZEN 3690, Malvern Instruments Inc., Malvern, UK).

### 2.7. Rheological Properties 

Using a DHR-1 hybrid rheometer (MCR302, Anton Paar, Austria) equipped with a parallel plate (60 mm in diameter and 1 mm in thickness) connected to a cooling system (Thermo Cube, New York, NY, USA). The flow behavior of the samples was determined using tests by Zhao, Wang, Zou, Li, Kang, Xu and Zhou [21]. The apparent viscosity was determined in the shear rate range of 0.1–1000.0 s^−1^ at 25 °C with a gap width of 1000 μm. The range of 1–10 s^−1^ was selected to ensure that any changes could clearly be observed. Additionally, we analyzed to determine the storage modulus (G′) and loss modulus (G″) as a function of the angular frequency (ω) in the range of 0.1–100.0 rad/s. The range of 1–10 rad/s was selected to ensure that any changes could clearly be observed. The G′ profile of the WBMP was measured while the temperature was increased from 20 °C to 80 °C at a heating rate of 1 °C/min (thermal gelation), and the G′ values were recorded continuously.

### 2.8. Microstructure of the Emulsions

#### 2.8.1. Optical Microstructure

Fresh emulsion (1 mL) was diluted in 1 mL of PBS buffer (20 mmol/L NaH_2_PO_4_/Na_2_HPO_4_, 0.1 mol/L NaCl, pH 6.0). The microstructure of the emulsion was observed using an optical microscope (Carl Zeiss, scope A1, Nussloch, Germany) with 40 × objective lenses.

#### 2.8.2. Confocal Laser Scanning Microscope

A 40 × objective lens confocal laser scanning microscope (CLSM) (Leica, TSC, Sp8 X, Nussloch, Germany) was used to observe the microstructures of the samples. A certain amount of emulsion was diluted with two volumes of PBS buffer (CLSM operates in the fluorescent mode. In total, 20 mmol/L NaH_2_PO_4_/Na_2_HPO_4_, 0.1 mol/L NaCl, pH 6.0). The diluted samples were fully mixed with 40 μL Nile Blue (0.01%, *w*/*v*) and Nile Red (0.01%, *w*/*v*) solutions. After dyeing for 2 h, 80 μL of the stained sample was placed on a glass slide and covered with a cover glass to ensure that there was no air gap or bubbles between the mixture and cover glass. After mixing evenly for 2 min, images of the Nile Blue and Nile Red were taken at excitation wavelengths of 488 and 633 nm, respectively. Each sample was randomly captured five times (1024 × 1024 pixels each).

### 2.9. Cream Index of the Emulsions during the Cold Storage

The cream index (CI) of the emulsion was determined after a small amount of modification according to Shi, Liu, Song, Xiong, Yuan, McClements, Jin, Sun and Gao [22]. Then, 20 mL of the newly formulated emulsion was transferred to a single glass bottle with a plastic cap and stored at 4 °C to measure the CI. The serum layer height (H_1_, cm) and total emulsion height (H_0_, cm) were recorded every 12 h from 0 to 168 h after emulsion preparation. Equation (3).

(3) $$\mathrm{CI}=\frac{H_{1}}{H_{0}}\times 100\%$$

### 2.10. Measurement of Lipid and Protein Oxidation

#### 2.10.1. Lipid Oxidation

Lipid oxidation was detected by monitoring the formation of lipid hydrogen peroxide (the primary product) and 2-thiobarbituric acid reactive substances (TBARS, the secondary product). The lipid hydrogen peroxide content was analyzed according to the method described by [23]. In addition, the lipid hydrogen peroxide content was calculated using a cumene hydrogen peroxide calibration curve. The TBARS analysis was performed as previously described [23]. The sample (1 mL) was added to thiobarbituric acid (TBA) reagent (2 mL), which contained 150 g/L trichloroacetic acid (TCA), 3.75 g/L TBA, and 0.25 mol/L HCl. The mixture was boiled for 30 min, cooled to room temperature, and centrifuged at 2000× *g* for 15 min. After mixing, the absorbance of the water layer was measured at 532 nm using an ultraviolet–visible (UV–Vis) spectrophotometer and was allowed to stand for 10 min. The concentration was then calculated using a standard curve composed of 1,1,3,3-tetraethoxypropane.

#### 2.10.2. Protein Oxidation

The carbonyl groups were slightly modified according to previously reported methods [24]. An aliquot of the emulsion sample (5 mL) was added to 5 mL of TCA at 200 g/L. The mixture was incubated in an ice bath for 10 min and centrifuged at 12,000× *g* for 15 min. The collected WBMP emulsion was then redissolved in 10 mL of sodium dodecyl sulfate (20 g/L, pH 8.0). Degreased hexane was added to 5 mL of the emulsion sample, and the solution was centrifuged at 12,000× *g* for 15 min. The carbonyl content was calculated using Equation (4).

(4) $$\mathrm{Carbonyl}\mathrm{content}\left(\mathrm{nmol}/\mathrm{mg}\right)=\frac{{3\times A\times 10}^{6}}{C\times V\times 22000}$$

where A is the absorbance, C is the protein concentration (mg/mL), and V is the protein volume (mL).

The sulfhydryl content was determined using a previously described method [23]. The absorbance was recorded at 370 nm, and the free sulfhydryl content was determined using a UV–Vis spectrophotometer. The blank was run with 10 mM phosphate buffer (pH 3.0 and 7.0). The sulfhydryl content was calculated using Equation (5) with a molar extinction coefficient of 13,600 M/cm.

(5) $$\mathrm{Sulfhydryl}\mathrm{content}\left(\mathrm{nmol}/\mathrm{mg}\right)=\frac{{A\times 10}^{5}}{136\times C}$$

where A is the absorbance and C is the protein concentration (mg/mL).

### 2.11. Statistical Analysis 

One-way and two-way analysis of variance (ANOVA) was used to determine the statistical differences. Multiple comparisons were performed according to Duncan’s multiple range test using the SPSS19.0 software (SPSS Inc., Chicago, IL, USA). All values were expressed as mean ± standard deviation with a significant difference of *p* < 0.05.

## 3. Results and Discussion

### 3.1. Physicochemical Properties of WBMP Emulsions

#### 3.1.1. Emulsifying Properties and ζ-Potential

**Figure 1:**
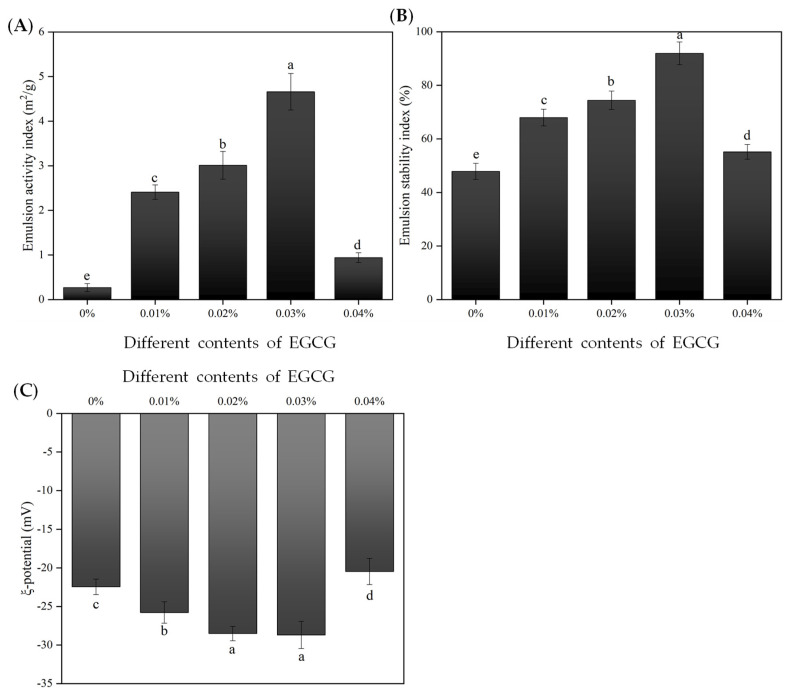
Changes in the emulsifying ability index (**A**) and emulsifying stability index (**B**). Determination of the ξ-potential of emulsions containing different levels of (-)-epigallocatechin-3-gallate (EGCG) (**C**). Means with different letters (a–d) within the same parameter group differ significantly (*p* < 0.05). Note: Control (0%): emulsions prepared by wooden breast myofibrillar protein (WBMP) alone; 0.01%-0.04%: emulsions prepared by WBMP with the addition of 0.01%, 0.02%, 0.03%, or 0.04% (*w*/*v*) EGCG, respectively.

The EAI reflects the formation of emulsions and the adsorption capacity of proteins at the oil/water interface. ESI refers to the ability to prevent phase separation (resist separation phenomena such as emulsion stratification, flocculation, and precipitation) and maintain emulsion stability [25]. The changes in EAI and ESI in each sample group are shown in Figure 1. Compared to the control group (without EGCG), the addition of EGCG significantly (*p* < 0.05) enhanced the EAI and ESI of the WBMP emulsion. When the addition of 0.01% EGCG, the EAI and ESI of the WBMP emulsion observably (*p* < 0.05) increased from 0.27 ± 0.09 m^2^/g and 47.88 ± 2.99% to 2.41 ± 0.16 m^2^/g and 67.97 ± 3.10%, respectively. Moreover, when the EGCG content was further increased to 0.03%, the EAI and ESI of WBMP emulsion increased to a maximum of 4.66 ± 0.41 m^2^/g and 91.95 ± 4.23%, respectively. We theorize that the combination with EGCG enhances the hydrophilicity of the protein surface and reinforces its interfacial activity. Furthermore, the adsorption speed of the oil–water interface improved and was conducive to the formation of dense and thick emulsion bases [23]. The same report confirmed that the improvement in the emulsifying performance was due to the increase in protein flexibility, solubility, and surface hydrophobicity, which improves the adsorption capacity of proteins on oil–water surfaces [26]. However, when the addition of EGCG increased to 0.04%, the EAI and ESI of WBMP emulsion significantly (*p* < 0.05) decreased (0.94 ± 0.11 m^2^/g, 55.16 ± 2.74%). It is suspected that the high concentration of EGCG affects the thickness of the interfacial film, resulting in the slight coalescence and flocculation of the emulsion [27]. Cheng, Zhu and Liu [6] also reported that the effect of polyphenol addition on the thickness of the protein emulsion interfacial film was critical to the EAI and ESI values. The concentration of EGCG had a decisive impact on the emulsifying performance of the WBMP, and an appropriate amount (0.03%) of EGCG observably enhanced the EAI and ESI of the WBMP emulsion.

**Figure 2:**
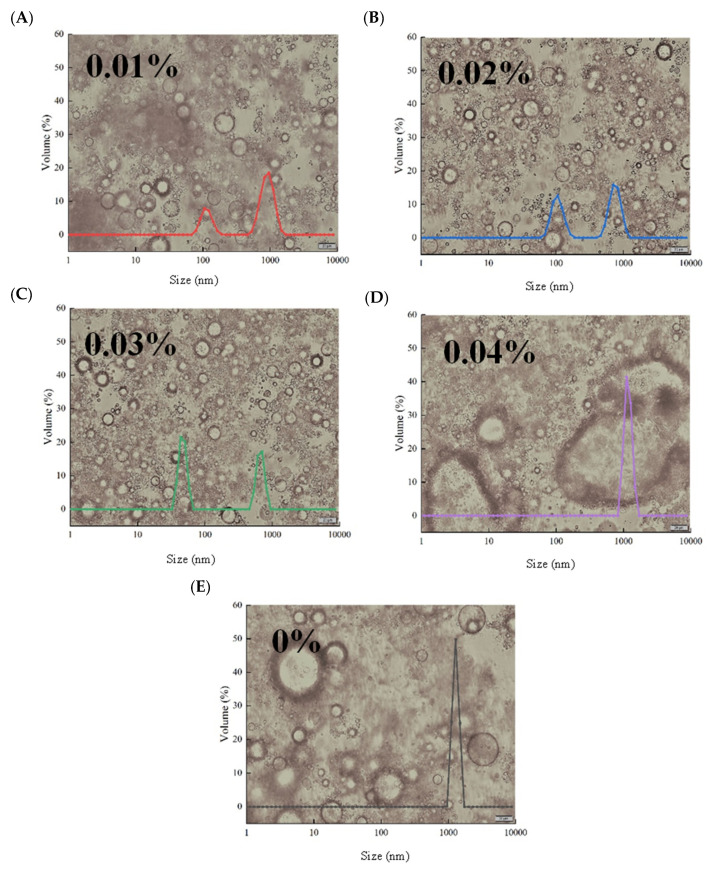
Changes in droplet size distribution and optical micrograph of wooden breast myofibrillar protein emulsion with different (-)-epigallocatechin-3-gallate addition (**A**–**E**). Note: intermediate peak A (around 100 nm), intermediate peak B (around 1000 nm).

The charge intensity carried by the emulsion droplets is very important for the stability and function of the emulsions [28]. It can be seen in Figure 1C that the charge of droplets can be significantly (*p* < 0.05) increased after adding EGCG, in which 0.01% EGCG increased the ζ-potential from −22.45 ± 1.01 mV to −25.78 ± 1.38 mV. Moreover, when the addition amount reached 0.03%, the potential accumulated to the maximum (−28.69 ± 1.77 mV). This is because the binding of anionic polyphenols (such as EGCG) to proteins increases the absolute value of the charge [29]. The increase in ζ-potential is consistent with the increase in emulsion physical stability because of the electrostatic repulsive force [16], which is consistent with the results of the WBMP emulsion particle distribution shown in Figure 2. Interestingly, when the EGCG content increased to 0.04%, the absolute value of ζ-potential decreased significantly (*p* < 0.05) in the initial emulsion (−20.47 ± 1.69 mV). A lower polyphenol content enhances the absolute value of charge, but higher levels reduce it [30]. This could potentially be due to the high content of polyphenols (such as EGCG) changing the position of the charged groups. 

#### 3.1.2. Droplet Size and Optical Microscope

Particle size often affects the surface properties and apparent structure of emulsions [16]. Figure 2 shows the particle distribution curve of the combined group of optical micrographs. The results show that the particle size of the emulsion droplets can be significantly (*p* < 0.05) reduced by adding EGCG. Figure 2 also shows the droplet dispersion and aggregate size in the optical micrograph. The size of the WBMP emulsion without EGCG was mainly distributed in the range of 955–1480 nm. The combination of protein and polyphenols has a favorable stabilizing effect on droplet aggregation and coalescence [31]. Optical microscopy showed that the original WBMP emulsion distribution was uneven, and there were more aggregates. When EGCG was added, the particle size distribution of the WBMP emulsion was significantly improved. When the amount of EGCG was 0.01%, the particle size of the WBMP emulsion increased from 955–1480 nm to 531–1480 nm, and a new peak appeared at 78.8–190.0 nm, which represented the droplet developing towards smaller particles at this time. However, the particle size change of 0.02% EGCG was not obvious, indicating that the amount of addition was not sufficient to cause the size and distribution of WBMP emulsion droplets to change abruptly. However, after adding 0.03% EGCG, the particle size distribution of the WBMP emulsion decreased to 37.8–58.8 nm and 531–825 nm. Liu, Wang, Sun, McClements and Gao [32] also proved the average diameter. The optical micrographs of 0.03% EGCG show that WBMP emulsion particles have a smaller particle size and more even dispersion than the other groups. However, it should be noted that when the amount of EGCG increased to 0.04%, flocculation and aggregation of the WBMP emulsion occurred, and the particle size distribution was mainly 955–1480 nm. It has been suggested that the excessive addition of EGCG may lead to its interaction with WBMP or hydrophobic interactions, resulting in a larger droplet size [33]. The above data indicate that adding 0.03% EGCG can improve the physical stability of the WBMP emulsions.

#### 3.1.3. Rheological Properties

**Figure 3:**
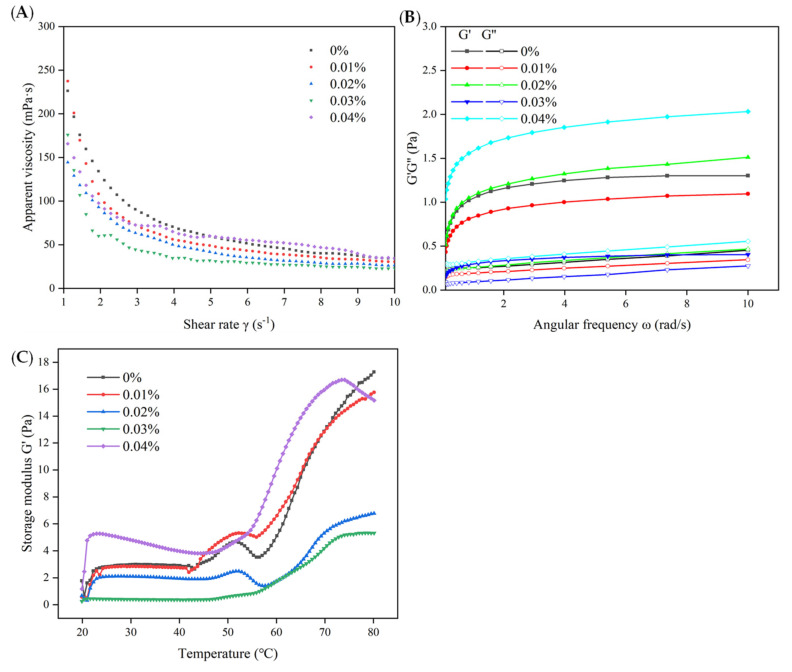
Flow curves (viscosity versus shear rate) (**A**), plots of G′ and G″ versus ω (**B**) and changes in G′ (**C**) of wooden breast myofibrillar protein emulsion heated from 20 to 80 °C with different (-)-epigallocatechin-3-gallate additions.

The viscosity of an emulsion can generally be seen when the difference between emulsions decreases with an increase in the shear rate (Figure 3A), which is due to structural fracture and rearrangement caused by the shear force. According to the overall trend of emulsion viscosity in Figure 3A, the apparent viscosity of the emulsion first decreased and then increased with the addition of EGCG from 0% to 0.04%. Among them, 0% was the control group, and its viscosity was markedly higher than those of the other groups. This could be closely related to the particle size of the droplets, as the particle size of the control group and the 0.04% EGCG group was significantly higher than that of the other groups (Figure 2). According to Zhao, Wu, Xing, Xu and Zhou [34], gravity drops are produced when the droplet size is large and the viscosity of the emulsion produced at the same rate of shear stress is greater. The protein and oil in the 0.03% EGCG group were well wrapped and distributed, so the resistance to shear was smaller, and the apparent viscosity became weaker accordingly. However, in the 0.04% EGCG group, the combination of high concentrations of EGCG and protein resulted in a more obvious aggregation and flocculation of the WBMP emulsion. The viscosity of the WBMP emulsion increased to a higher viscosity at the same shear rate.

**Figure 4:**
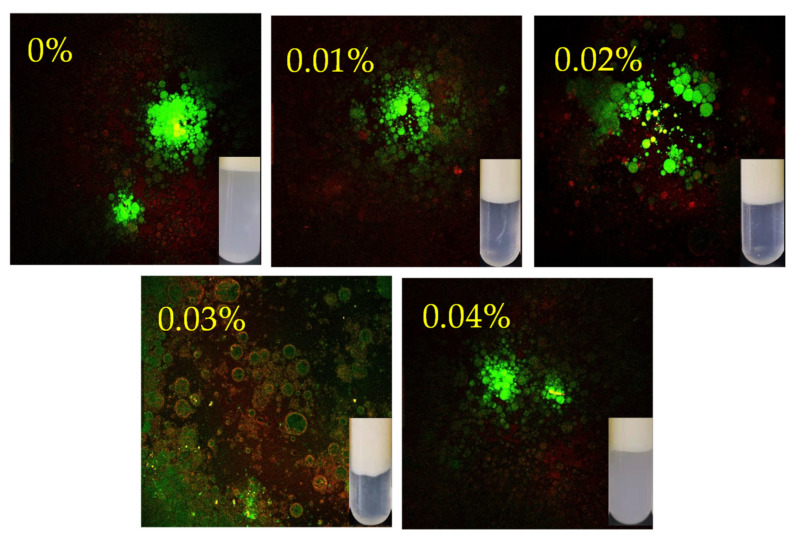
Changes in the confocal laser scanning microscopy micrographs and images after stabilization of wooden breast myofibrillar protein emulsions under different contents of (-)-epigallocatechin-3-gallate.

In general, a formed gel was stronger when there was a larger difference between the G′ and G″. However, this is not always advantageous in emulsion systems. The frequency sweep test (Figure 3B) showed that the G′ in each group was higher than the G″, and the trend was consistent within the test range without an intersection. These characteristics demonstrate that the structure is ordered and elastic. Similar to the apparent viscosity (Figure 4A) trend, the G′ of the control group was much larger than that of G″. This indicates that the oil in the emulsion is not integrated with WBMP, which is more conducive to the formation of WBMP gel and not to the uniform dispersion of the emulsion. The difference between the G′ and G″ in the 0.03% EGCG group was relatively small, indicating that the emulsion was a weak gel [35]. It was caused by the electronic interaction between the EGCG and WBMP. This association lowers the water-binding capacity of the WBMP, thus reducing the viscosity of the emulsion [9]. It also interferes with the formation of the protein network and reduces the G′ value to a certain extent. Interestingly, the addition of a high concentration of EGCG promotes the aggregation of WBMP, which results in the formation of a strong gel network [36]. Therefore, the G value of the 0.04% EGCG emulsion was much higher than that of G’, and the difference was significantly larger than that of the other groups.

The myosin heads expand and interact with each other to form an elastic network at approximately 48 °C, inducing an increase in the G′ value. Subsequently, the denaturation resulted in a significant increase in the G’ value until the end of cooling [34]. The rheological properties of the WBMP emulsions during heating are shown in Figure 3C. The control and 0.01% EGCG groups showed complete elastic gel changes related to myosin degeneration. The emulsion showed a more elastic solid behavior under these circumstances. This indicated that the individual WBMP was still in the dominant position and that the gel was formed during the heating process. The curve of the 0.03% EGCG group tended to be gentle at the first peak, indicating that the emulsion behaved as a viscous liquid [37]. We suspect that the uniform combination of protein and oil droplets hinders the formation of a network, resulting in the disappearance of the peak. von Staszewski, Jagus and Pilosof [38] also proved that the emulsion formed by the covalent interaction between the EGCG and the protein was more stable [39]. The peak of the curve in the 0.04% EGCG group was not obvious, but its G′ value was much higher than in the other groups. This suggests that the combination of high concentrations of EGCG and WBMP promotes protein aggregation and enhances the elastic properties of the emulsion [16]. The results confirmed that the 0.03% EGCG emulsion exhibited better viscous emulsion properties.

#### 3.1.4. Confocal Laser Scanning Microscopy

The oil is dyed green with Nile Red, and the protein is dyed red with Nile Blue (Figure 4). In the control group without EGCG, it can be found that large droplets are wrapped by an incomplete protein membrane, and many free protein masses are displayed. This is mainly because WBMP converged and cannot be dissolved, which makes it unable to migrate to the oil–water interface and form a membrane [16]. When EGCG was added, the droplet diameter began to decrease, and the protein distribution was more uniform. However, there were still some small and dense aggregates in the 0.01% and 0.02% groups. After the addition of 0.03% EGCG, the droplet diameter of the WBMP emulsion decreased, the protein film was wrapped tightly, and other proteins were evenly dispersed in the water phase. Similarly, when the EGCG was further increased to 0.04%, obvious local aggregation occurred. The formed structure was relatively loose and composed of droplets attracted by protein aggregates. The physical stability of the emulsion is dependent on the combination of EGCG and protein. However, excessive EGCG causes the aggregation of emulsion droplets owing to the location of the charged groups [16]. This confirmed the optical observations and the particle size results. We believe that the main reason for protein aggregation caused by a high concentration of EGCG is that 0.04% EGCG promotes the oxidation of WBMP, which is also reflected in the protein oxidation index we determined later. Therefore, the hydrophobic interaction and electrostatic interaction of proteins are also affected, and gradually, aggregation occurs. Among them, WBMP(control group) is a good example, because the WBMP particles become larger and aggregate due to protein oxidation. Additionally, a high concentration of EGCG counteracts the good effect of polyphenols on protein structure improvement, which promotes the aggregation of protein film-wrapped droplets in the emulsion. This research result is reflected in Tian, Kejing, Zhang, Yi, Zhu, Decker and McClements [16].

#### 3.1.5. Cream Index

**Figure 5:**
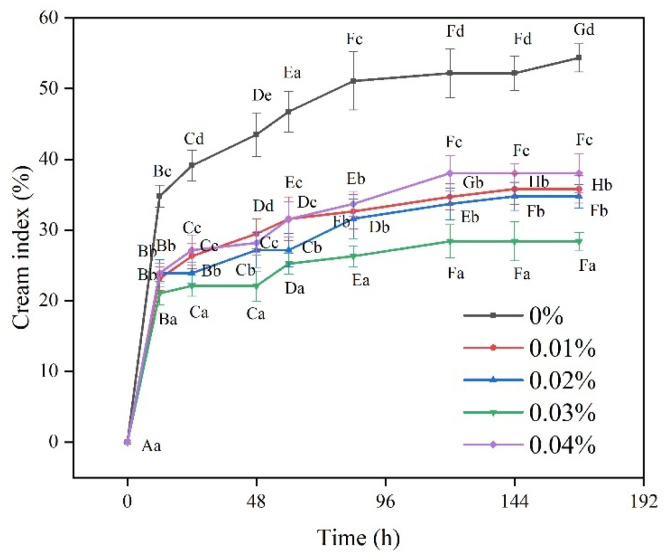
Cream index of wooden breast myofibrillar protein emulsions with different (-)-epigallocatechin-3-gallate contents. Means with different letters (a–e, A–G) within the same parameter group differ significantly (*p* < 0.05).

The CI represents the change in emulsion stability during storage. Figure 5 shows the changes in each CI group during the 7 d (day) storage process. The CI of the WBMP emulsion decreased significantly after the addition of EGCG, especially in the 0.03% EGCG group (*p* < 0.05). The main factors leading to the change in CI are the changes in gravity and density. The addition of EGCG reduced the CI of emulsions during storage. This is because the weak network formed by the EGCG and the protein fixed the local structure, which greatly impeded droplet movement [39]. The 0.03% EGCG group exhibited slow but continuous phase separation throughout the storage process. From the long-term change in the CI, 0.03% EGCG had more stable storage than the other groups. This showed that the binding energy between the 0.03% EGCG and the protein was more entangled with the droplets. Electrostatic repulsion, steric hindrance, and thickening effects are the main reasons for this [40], as proven by potential and microscopic images. Kanakis, Hasni, Bourassa, Tarantilis, Polissiou and Tajmir-Riahi [41] found that hydrophobic interactions between proteins and polyphenols could change the structure and interface of proteins. This property is conducive to the emulsion’s stability [42]. This temporary instability can become permanent through mergers and/or Ostwald maturity. Consequently, the emulsion tends to return to its original two-phase state. A protein–polyphenol complex is added to the emulsion in the form of an emulsifier and is adsorbed onto the surface of the emulsion droplets to form a dense membrane. Therefore, emulsion instability caused by coalescence or maturation of Ostwald can be prevented [43]. Similarly, 0.04% EGCG leads to WBMP aggregation owing to its high concentration, which makes it seriously stratified after long-term storage. It can be observed that the amount of EGCG should be controlled.

### 3.2. Effect of EGCG on Oxidative Stability of WBMP Emulsions

#### Lipid Oxidation and Protein Oxidation

**Figure 6:**
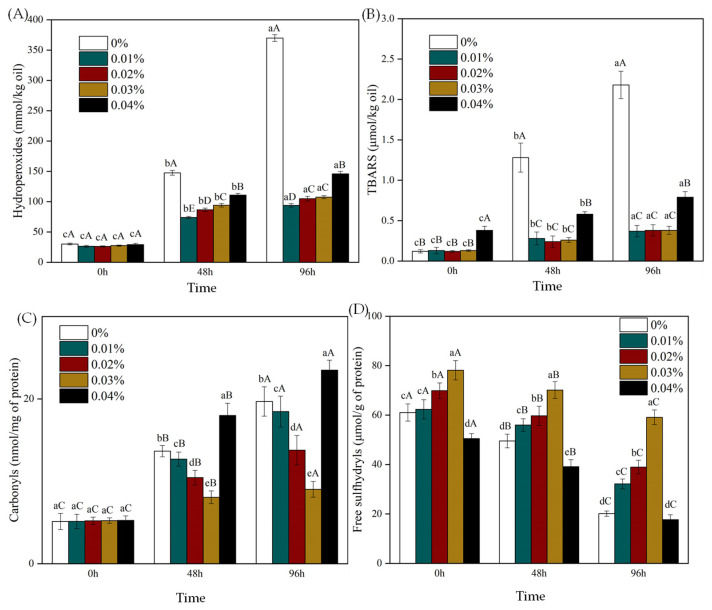
Generation of lipid hydroperoxides (**A**), thiobarbituric acid reactive substances (**B**), protein carbonyls (**C**), and free sulfhydryls (**D**) in emulsions containing different levels of (-)-epigallocatechin-3-gallate during storage. Means with different letters (a–d, A–E) within the same parameter group differ significantly (*p* < 0.05).

The oxidative degradation of lipids in food is a key factor that limits their shelf life and acceptability [24]. Therefore, after incubation at 50 °C for 0, 48, and 96 h, the effects of EGCG on the primary (lipid hydrogen peroxide) and secondary (TBARS) oxidation products of the lipid oxidation resistance of the emulsion were studied. The amount of lipid hydrogen peroxide gradually increased with increasing storage time, indicating the oxidative degradation of unsaturated lipids (Figure 6A). The addition of EGCG indicates the excellent antioxidant properties of EGCG. The efficacy of the highest dose used (0.04%) was lower than that of the lower doses (0.01%, 0.02%, and 0.03%). After being treated with 0%, 0.01%, 0.02%, 0.03%, and 0.04% EGCG for 96 h, the content of lipid hydrogen peroxide is 369.95 ± 5.47 mmol/kg oil, 93.78 ± 2.97 mmol/kg oil, 104.89 ± 3.88 mmol/kg oil, 107.48 ± 2.47 mmol/kg oil, and 145.89 ± 4.12 mmol/kg oil, respectively. This result indicates that the addition of polyphenols must be optimized to be as effective as possible. This is advantageous because fewer antioxidants are added to the system. The active groups in EGCG can scavenge free radicals by contributing their hydrogen atoms to form reactive phenoxy groups [44]. More importantly, the complex formed between protein and EGCG can enhance the antioxidant activity of WBMP and significantly improve its ability to scavenge free radicals [45]. However, we found that excess EGCG caused it to change from an antioxidant to an oxidative factor. This has also been confirmed in previous studies [46]. This could be due to endogenous iron increasing in the form of Fe^2+^ [47]. The effect of EGCG addition on TBARS formation (Figure 6B) was similar to that of lipid hydrogen peroxide (Figure 6A). The addition of EGCG significantly reduced the production of secondary reaction products during storage (*p* < 0.05), confirming the antioxidant effect of EGCG in the emulsion. EGCG can form a thick film on the surface of oil drops, which can prevent oil droplet oxidation and improve the stability of the emulsion [7]. The antioxidant activity of EGCG decreased with increasing concentration. Taking the emulsions with EGCG contents of 0%, 0.01%, 0.02%, 0.03%, and 0.04% as examples, the TBARS concentrations after 96 h were 2.18 ± 0.17 μmol/kg oil, 0.37 ± 0.07 μmol/kg oil, 0.38 ± 0.07 μmol/kg oil, 0.38 ± 0.05 μmol/kg oil, and 0.79 ± 0.07 μmol/kg oil, respectively. We demonstrated that EGCG could effectively inhibit lipid oxidation in the WBMP emulsion system. Therefore, an excessive concentration of EGCG promotes lipid oxidation and affects the storage properties of emulsions.

The chemical reaction substances produced by lipid oxidation may promote the oxidation of proteins. Polyphenols (EGCG) can combine with proteins to change their structure and surface chemistry properties to change their oxidation sensitivity [26]. We determined the effect of EGCG on protein oxidation in emulsions. The two most significant modifications of carbonyl and sulfhydryl derivatives after protein oxidation are the acquisition of carbonyl groups and the loss of sulfhydryl groups [48]. However, the addition of EGCG changed the degree of protein oxidation in the emulsions. The addition of 0.03% EGCG significantly reduced carbonyl formation (*p* < 0.05) compared to the 0.01% and 0.02% groups (Figure 6C), indicating that this level inhibited protein and lipid oxidation. The inhibitory effect of EGCG on protein carbonylation might be related to its ability to scavenge free radicals and chelate transition metal ions [49]. They can bind to protein molecules and produce phenolic protein complexes. However, increasing the concentration of EGCG in the emulsion will lead to increased carbonyl formation, indicating that higher concentrations promote protein oxidation. Taking 0%, 0.01%, 0.02%, 0.03%, and 0.04% EGCG as examples, the carbonyl contents in the emulsion after 96 h storage were 19.69 ± 1.78 nmol/mg of protein, 18.46 ± 1.88 nmol/mg of protein, 13.78 ± 1.78 nmol/mg of protein, 9.03 ± 0.96 nmol/mg of protein, and 23.48 ± 1.22 nmol/mg of protein, respectively. Protein oxidation is promoted at high doses (e.g., 200 μM) [50]. The EGCG concentration also affected the degree of sulfhydryl consumption in the emulsion (Figure 6D). The sulfhydryl contents in the emulsions of 0%, 0.01%, 0.02%, 0.03%, and 0.04% EGCG were 20.11 ± 1.06 μmol/g of protein, 32.15 ± 1.99 μmol/g of protein, 38.96 ± 2.78 μmol/g of protein, 59.12 ± 2.98 μmol/g of protein, and 17.69 ± 1.98 μmol/g of protein, respectively. This result showed that appropriate doses of EGCG (0.03%) had the highest antioxidant capacity, which was consistent with the measurement of carbonyl formation (Figure 6C). EGCG inhibits lipid oxidation more effectively than protein oxidation [16]. Among them, 0.03% EGCG had a more advantageous effect on fat and protein oxidation. However, when the addition of EGCG increased to 0.04%, the antioxidant effect was greatly decreased and further promoted oxidation. This could be due to the presence of high concentrations of polyphenols enhancing the binding of transition metals to the protein surface [51].

### 3.3. Effect of Different EGCG Content on WBMP Emulsion Droplets

**Figure 7:**
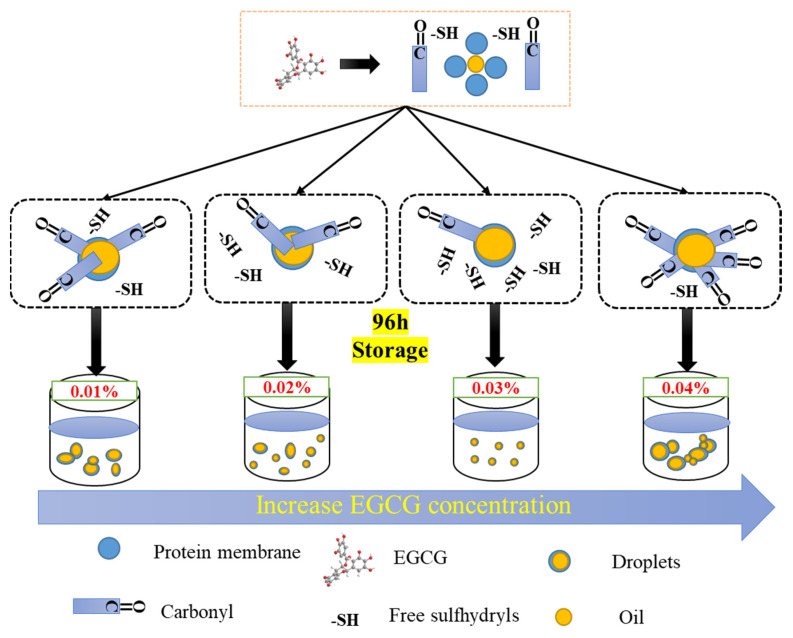
Schematic diagram of the effect of EGCG with different contents on WBMP emulsion.

In order to more clearly show the role of EGCG with different concentrations in the formation of WBMP emulsions, we show its preliminary mechanism in Figure 7. It can be seen from the figure that the emulsion formed by WBMP without any treatment has a large particle size and a lot of aggregation, so it cannot be stored for a long time. The addition of EGCG greatly improved this phenomenon. Many droplets began to become smaller and could be more evenly dispersed. However, contents of 0.01% and 0.02% still could not meet the quality improvement of the emulsion, and there were still uneven droplets distributed in the emulsion. When the content of EGCG reaches 0.03%, the protein film formed by EGCG and protein can wrap small droplets well and can be uniformly dispersed without aggregation due to the increase in electrostatic repulsion. However, when a high concentration of EGCG (0.04%) is added, the hydrophobic interaction of the protein starts to increase, while the surface charge starts to decrease, which leads to protein aggregation, and this concentration also starts to promote the oxidation of the protein, leading to the large-scale aggregation of emulsion droplets, and the oxidation is so serious that it is not conducive to preservation. The change of the active sulfhydryl group content and the carbonyl group content representing the oxidation state also explained the influence of EGCG on the antioxidant properties of the WBMP emulsion after storage. This is also the main reason why different concentrations of EGCG exist in the emulsion, leading to the dispersion/aggregation of its emulsion droplets.

## 4. Conclusions

The effects of different amounts of EGCG on the physicochemical properties and oxidation stability of WBMP emulsions were studied. The difference in EGCG contents showed two opposite effects in improving the quality of WBMP emulsions. The results showed that low-dose (0.01%, 0.02%) EGCG did not bind protein well, so it did not improve the performance of the emulsion; 0.03% EGCG improved the emulsion performance, interfacial rheology, and physical stability of the WBMP emulsion (1.2% WBMP/10% oil) by protein interaction. The oxidation stability of emulsions was also promoted through the inhibition of lipid and protein oxidation. This is due to the complex formed by EGCG and WBMP, which encapsulates a layer of film for emulsion droplets and guarantees the stability of the emulsion. In contrast, higher EGCG concentrations (0.04%) resulted in protein over-aggregation in improving the quality and oxidation stability of the emulsion. In conclusion, EGCG can significantly improve the quality of WBMP emulsions. However, the amount of EGCG added should be controlled, too low or too high concentration cannot achieve good results. This also provides a theoretical basis for improving the use of inferior proteins, similar to WBMP. Similarly, these works may provide a reference for the application of EGCG in protein-based emulsions.

## Data Availability

Data are contained within the article.
